# Transposable Elements: Major Players in Shaping Genomic and Evolutionary Patterns

**DOI:** 10.3390/cells11061048

**Published:** 2022-03-19

**Authors:** Nunzia Colonna Romano, Laura Fanti

**Affiliations:** Istituto Pasteur Italia, Dipartimento di Biologia e Biotecnologie “Charles Darwin”, “Sapienza” University of Rome, 00185 Rome, Italy; nunzia.colonnaromano@uniroma1.it

**Keywords:** transposable elements, environmental stress, evolution, epigenetics

## Abstract

Transposable elements (TEs) are ubiquitous genetic elements, able to jump from one location of the genome to another, in all organisms. For this reason, on the one hand, TEs can induce deleterious mutations, causing dysfunction, disease and even lethality in individuals. On the other hand, TEs can increase genetic variability, making populations better equipped to respond adaptively to environmental change. To counteract the deleterious effects of TEs, organisms have evolved strategies to avoid their activation. However, their mobilization does occur. Usually, TEs are maintained silent through several mechanisms, but they can be reactivated during certain developmental windows. Moreover, TEs can become de-repressed because of drastic changes in the external environment. Here, we describe the ‘double life’ of TEs, being both ‘parasites’ and ‘symbionts’ of the genome. We also argue that the transposition of TEs contributes to two important evolutionary processes: the temporal dynamic of evolution and the induction of genetic variability. Finally, we discuss how the interplay between two TE-dependent phenomena, insertional mutagenesis and epigenetic plasticity, plays a role in the process of evolution.

## 1. Introduction

The history of the evolutionary thought is an exciting story. Although the Darwinian theory of evolution by natural selection is now commonly accepted, it is still rich in implications and facets that define all its possible mechanisms. One point of debate is the temporal dynamic of evolution. Evidence suggests that evolution is continuous, yet it does not proceed at a constant speed. This notion is supported, on the one hand, by the presence of species that have remained substantially unchanged over millions of years, and on the other hand, by cases of rapid evolution. Additionally, from a paleontological point of view, evolution is evidenced by the abrupt appearance of new variants, followed by stasis, before a new explosion of life forms emerge again [1,2,3,4,5,6,7]. This concept was originally proposed by Cuvier in the 1790s [8], and subsequently re-elaborated [9,10,11] in the theory of punctuated equilibrium [12,13]. This view, applied to a biological scale, is not strictly consistent with neo-Darwinism. Instead, the hypothesis by McClintock [14], that the production of new species occurs by ‘saltation generation’, fully falls within this idea. According to McClintock’s theory, transposable elements (TEs), jumping from one part of the genome to another, create mutations that increase genetic variability and eventually induce morphological alterations that allow adaptation to environmental change.

**Figure 1:**
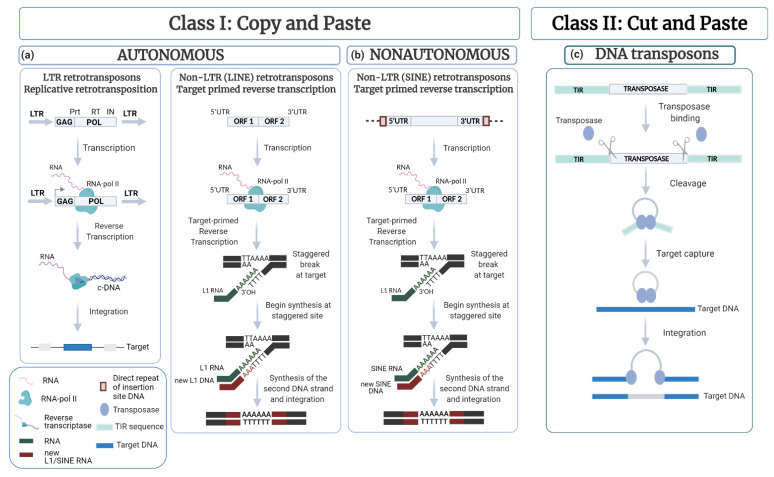
Schematic representation of the different mechanisms of transposition. Generally, TEs can be distinguished in two major classes on the basis of their mechanism of transposition: active eukaryotic Class I (retrotransposons) and active eukaryotic Class II (DNA transposons). Additionally, TEs can be divided into autonomous and non-autonomous. (**a**) Class I (retrotransposons) require RNA transcription to be able to move to different genome locations. They encode for a reverse transcriptase enzyme that uses the transcript as a template to produce a cDNA sequence that reinserts randomly into a new genomic site. This is the so-called “copy and paste” mechanism. Autonomous Class I RNA transposons encode all proteins necessary for moving. They include long terminal repeats/endogenous retroviruses (LTR/ERV; e.g., the yeast *Ty* element) and non-LTR retrotransposons such as the long interspersed nuclear elements or LINEs (e.g., human L1). LTR-retrotransposons contain two long terminal repeats (LTRs, grey arrows) and genes encoding for functional proteins, such as *Gag* (*group-specific antigen*), *Pol* (*reverse* *transcriptase*), *Int* (*integrase*) and *Prt* (*protease*). The non-LTR retrotransposons also contain genes encoding for enzymes required for transposition but lack LTRs. Instead, they have two open reading frames flanked by a 5′ and a 3′ untranslated region (UTR). Generally, these TEs mobilize by a target-site primed reverse transcription (TPRT) mechanism. After the hydrolysis of one strand of DNA at a new insertion site, the 3′OH end of this strand is used to prime the reverse transcription of a new LINE cDNA by the reverse transcriptase encoded by the element. Subsequently, hydrolysis of the second DNA strand releasing a 3′OH end that primes replication of the second strand of the LINE cDNA. Finally, the integrase completes the insertion. (**b**) Non-autonomous retrotransposons rely on “true” (autonomous) retrotransposon activity for mobility. For example, SINE elements (like *Alu*) have an internal promoter for RNA polymerase III flanked by a 5′ and a 3′ UTR but lack genes encoding enzymes required for transposition. SINEs use the same TPRT mechanism to transpose, but they must borrow the necessary activity from LINE to insert. (**c**) Class II (DNA transposons) encode the protein transposase (TPase) flanked by terminal inverted repeats (TIRs). TPases are responsible for removing and inserting TEs in a new genomic location according to two different mechanisms. One is the so-called “cut and paste” or “non-replicative pathway” mechanism through which a TE is excised from its locus and reinserted at another site. The second is the “replicative pathway” in which a TE is copied, and the copy is relocated, leaving behind the original.

TEs belong to several families, which differ in structure and in the modality of transposition. TEs make up about half of the human genome, and about 85% of the maize genome [15,16]. There are several types of TEs [17,18] but they can be divided into two major classes, depending on the mechanism of transposition. Class I contains the so-called *retrotransposons* elements, which move via reverse-transcribed RNA sequences that integrate into the genome. They are represented by long terminal repeat/endogenous retrovirus (LTR/ERV) elements, non-LTR retrotransposons (such as long interspersed nuclear elements or LINEs) and non-autonomous elements (such as short interspersed nuclear elements or SINEs). Class II elements mobilize via a DNA intermediate, either by a cut-and-paste mechanism, catalyzed by transposase enzymes, or by rolling-circle DNA replication (helitrons) [19], or by other unknown mechanisms (polinton/mavericks) [20]. This class also includes non-autonomous TEs, such as truncated DNA transposons and miniature inverted-repeat TEs (MITEs) [17,21] (Figure 1). The presence of transposons within genomes is dynamic [22,23,24]. TEs tend to increase in number via transposition but can also accumulate mutations, leading to their extinction. However, TEs are widespread in the genome of all organisms. This is because at least some TEs remain active and maintain the capacity to invade new species through horizontal transfer [25,26].

## 2. TEs as Parasites of the Genome

The transposition of TEs can be deleterious for the host, as this can induce gene mutation by insertional mutagenesis and chromosomal rearrangements [18,27,28,29,30]. TEs that move with a cut-and-paste mechanism can generate frame shift mutations by inserting into coding sequences or by causing small deletions through imprecise excision. Additionally, TEs can induce chromosomal inversions or large deletions/duplications, following recombination between different copies of the same element. Furthermore, more complex chromosomal rearrangements can result from alternative transposition events when complementary TE ends from separate TEs form a hybrid element on sister chromatids. This mechanism could produce a dicentric chromosome and an acentric fragment [27,31]. For this reason, TEs have been considered genomic parasites that exploit the host machinery for their maintenance and propagation. In line with their parasitic origin and selfish behavior, TEs have long been associated with mutant phenotypes and diseases, in both humans and animals. For example, hybrid dysgenesis is a complex syndrome, discovered in *Drosophila* [32,33], and caused by the mobilization of P-elements in crosses between males that carry these TEs and females that lack them. This syndrome is characterized by germline abnormalities, frequent mutations, and chromosome breakages. However, these abnormalities are not seen in the reciprocal crosses because of the presence of a ‘cytoplasmic factor’ in the female germline that prevents TEs’ mobilization.

**Figure 2:**
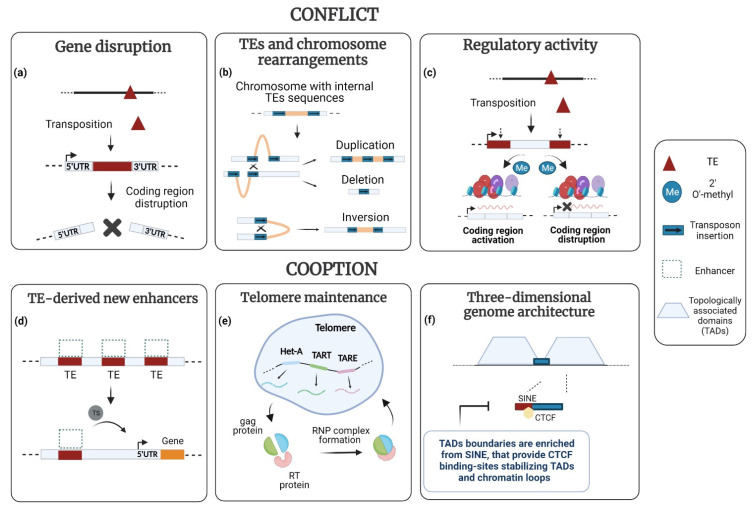
TEs as ‘molecular parasites’ or ‘functional symbionts’. As ‘molecular parasites’ TEs can produce a variety of detrimental effects on the host genome. (**a**) The insertion of TEs within coding exons can cause frame shift mutations disrupting protein sequence and function. (**b**) TEs can cause genomic instability being the substrate for chromosome rearrangements, such as duplications, deletions, inversions and translocations. (**c**) The insertions of TEs in regulatory stretches such as in 5′ or 3′ regions or introns can cause epigenetic modifications resulting in inappropriate activation or repression of gene expression. The co-option of TEs by the host genome may generate new regulatory signals or coding sequences. This process is referred to as ‘molecular domestication’. (**d**) TEs may contribute new enhancer sequences for transcription factors (grey circle) changing the spatial/temporal regulation of gene expression. (**e**) After the loss of telomerase, retrotransposons can actively participate in the maintenance of telomeres. Three non-LTR families, HeT-A, TAHRE, and TART form a head-to-tail array. They express Gag and Reverse Transcriptase proteins that are necessary for the elongation of telomeres. (**f**) TEs can contribute to the maintenance of genome architecture by providing binding sites for the CTCF protein that is responsible for establishing “topologically associated domains” (TADs). “Created with BioRender tool. https://app.biorender.com/” (accesed on 8 February 2022).

*De novo* germline TE insertions that disrupt normal gene function have been implicated in more than one hundred human inherited diseases [34,35] (Figure 2a). For instance, LINE-1 retrotransposons are, themselves, responsible for at least 25 reported cases of human illness, including Duchenne muscular dystrophy, hemophilia B, β-thalassemia trait, and chronic granulomatous disease [36]. LINE-1 retrotransposition can disrupt coding exons or occur into introns [37], which may induce exon skipping or mis-splicing and lead to the generation of null or hypomorphic alleles. As an example, a SINE-VNTR-*Alu* (SVA) insertion into the *fukutin* gene results in abnormal splicing and in the development of Fukuyama muscular dystrophy (FCMD). Fukutin is a putative transmembrane protein localized to the cis-Golgi compartment and found at high levels in skeletal muscle, the heart and brain [38]. Additionally, some evidence suggests an intricate association between TEs de-repression and ageing [39,40,41]. Indeed, the weakening of defense mechanisms with age contributes to the de-repression of retrotransposons and, thus, to several age-related diseases [42,43]. Evidence suggests that retroelements can reactivate in senescent cells, with direct repercussions on longevity [44]. A clear demonstration comes from *Sirt-6*-deficient mice and flies, showing greatly improved longevity after Nucleoside Reverse Transcriptase Enzyme Inhibitor (NRTI) treatment [45].

The mobility of TEs can induce genomic instability [34,46,47,48] (Figure 2b). In somatic cells, both transposition and TE-mediated inter- and intra-chromosomal rearrangements appear casually linked to several types of cancer [35,49,50,51,52,53,54]. Possibly, the human DNA transposon *Tigger 1* is the cause of a new deletion/insertion mutation in the *BRCA-1* gene, related to cancers of the breast and ovaries. An excision event of *Tigger 1* would have caused the deletion of some nucleotides in the *BRCA-1* gene that have become incorporated into the sequence of the terminal inverted repeats (TIRs) of the transposon [49]. In Hodgkin Lymphoma cell lines, the pro-inflammatory factor *IRF5* is upregulated, following the activation of an *LTR-IRF5* chimeric transcript. This can give rise to a singular mechanism of oncogene activation, named “onco-exaptation”, widely studied in cancer [51]. Interestingly, Human endogenous retroviruses (HERVs) seem involved in the remodeling of transcriptional networks. For example, the upregulation of the oncogenes *ETV1* and *CSF1R* causes prostate cancer and glioblastoma, respectively. These effects allegedly depend upon *HERV LTR* producing chromosomal rearrangements in the former and cis promoter activation in the latter [53]. In addition, mutations of p53, a protein involved in cellular stress responses and apoptosis, can lead to transposition and to genomic instability in cancerous cells [55].

## 3. TEs as Symbionts within the Host Genome

As mentioned above, transposons can modify gene expression and impact regulatory networks through their insertion into functioning genes. However, the majority of new TE insertions are not harmful and are not selected against. Thus, they may persist in a population as polymorphisms, and genetic drift may drive a subset of them to become common alleles or to becoming fixed in a species.

There is growing evidence that TEs are inducers of biodiversity, having acquired important functions during evolution [56,57]. In this context, transposons can also be considered ‘symbionts’ of the genome.

### 3.1. TEs and New Regulatory Programs

TEs can act at various levels: gene, chromatin and chromosomal. For example, the host organism can benefit from abrupt new regulatory programs, emerging from new integration events that provide additional enhancers, alternative promoters, silencers [56,58,59,60,61,62,63] or the creation of new exons that add useful functions to gene products [64,65,66,67,68] (Figure 2c,d).

In the last few years, evidence has shown that TEs display the hallmark of active regulatory elements [69,70,71,72,73]. In fact, if, on the one hand, transposons are a source of disease, on the other hand, they are also the source of numerous and valuable regulatory sequences, recruited by “molecular domestication” [74,75]. These sequences have facilitated the evolution of further complexity in the regulation of transcription, and as such, components with contradictory effects may be involved. For example, the tumor suppressor p53 protein is responsive to DNA damage and cell stress signals, mainly through its transcription factor activity. Many p53 DNA-binding sites are highly enriched in ERV-LTR elements that impact the expression of p53 target genes and seem to contribute to the generation of species–specific regulatory networks [76]. In the fungal plant pathogen *Zymoseptoria tritici*, the insertion of TEs can create different patterns of expression of gene clusters engaged in melanin biosynthesis, through the formation of new epialleles [77]. The DNA transposon *mPing*, recently detected in several rice strains, is another interesting example of a TE acting as a master gene regulator. Preferentially, *mPing* targets the 5′-region of genes and is able to increase gene expression under conditions of stress [78]. This demonstrates that TEs can create new regulatory networks upon insertion, but can also modulate such networks in response to environmental stress. Tail loss in hominids and apes was mediated by the insertion of a single *Alu* element into the intron of the *TBXT* (*Brachyury*) gene, which, with a copy already present in the opposite orientation, resulted in the formation of a hairpin and an event of alternative junction [79,80].

In a long-term evolutionary perspective, TE-sequences reactivation (exaptation or co-option) could bring about advantages to the host, becoming coding and non-coding exapted TEs. More recently, genome sequencing is contributing new insights, facilitating the identification of exapted TEs that bring benefit to the host [81]. In fact, in addition to providing *cis*-regulatory elements, TEs can contribute to the production of a wide range of non-coding regulatory RNA transcripts, such as microRNAs (miRNAs) [82,83] and long non-coding RNAs (lncRNAs), which can modulate gene expression in *cis* or in *trans* [84,85]. For example, TE-derived ncRNAs have been coopted in the regulation and development of adaptive immunity, of the nervous system, and of the mammalian placenta. Indeed, some *envelope glycoprotein-encoding* (*env*) genes of Endogenous Retroviruses (ERV) have undergone a process of positive selection in different mammalian lineages, which has led to the expression of the *syncytin 1* and *2* genes, essential to the formation of the *syncytiotrophoblast* during placenta development [86]. The lncRNA *LincGET* and *lnc-RoR*, were derived from the *MERV-L* and the *HERV*-*RoR* loci, respectively. They both have a function as recruiters of transcription factors (TFs), involved in maintaining the pluripotent state of embryonic stem cells (ESC) in mice [87]. Novel insights on TFs’ occupancy and their ability to activate TEs, have provided interesting correlations between the co-option process of TEs and their impact on the regulation of gene expression of the host [88]. Often, TEs contain binding sites for TFs, through which they can recruit lineage-specific targets and regulate gene expression in specific tissues [89].

### 3.2. TEs and New Epigenetic Landscapes

TEs can also alter the epigenetic landscape. New insertions may cause local changes in DNA methylation or histone tail modifications, eventually affecting gene expression [90,91,92,93]. For example, in a recent study, Noshay et al. showed that in maize, the insertion of TEs into active regions of the genome is associated with an increased mutation load and abnormal histone tail modifications [94]. In fact, Slotkin and Martienssen have demonstrated that methylation of DNA and histone tail alterations, two classical marks of constitutive heterochromatin, suppress the activity of TEs [95]. These processes involve chromatin remodeling factors, such as KRAB-zinc finger proteins (KZFPs) in mammals [96,97,98] and DDM1 in plants [99]. The epigenetic silencing of TEs, by RNA-directed DNA methylation (RdDM), can expand to the promoter of neighboring genes and suppress their expression [100]. For instance, in the genome of maize, the transposon-mediated epigenetic downregulation of *ZmNAC111*, encoding for a widely expressed transcription factor, causes diminished resistance to drought [101]. Conversely, mutations in genes required to introduce ‘repressive’ histone tail modifications can lead to a significant reactivation of TEs, as evidenced in mice by a mutated *SUV39* gene encoding for a H3K9 (histone 3 lysine 9) methyltransferase [90]. Another interesting example of an interaction between TEs and chromatin modifications has been discovered in mice, where polymorphic copies of B2 SINEs serve as a boundary element that can modulate chromatin modifications and gene expression [102] (Figure 2c).

In the context of the symbiotic interaction between host and TEs, it has been argued that much of the adaptive epigenetic flexibility arose because of the need for eukaryotic genomes to control and domesticate parasitic TEs [103]. Evidence from epigenomic data suggests that some TE families have contributed to the evolution of tissue-specific gene regulatory networks in several contexts, such as early development [59,104,105,106,107], organogenesis [108,109,110], immunity [111], placentation [61] and pregnancy [60]. A good example in this regard is the DNA transposon (Class II TEs) *MER20*, which contains binding sites for various TFs, and chromatin signatures, associated with functional regulatory elements. *MER20* was found associated with more than 1500 differentially expressed genes in the stromal cells of the endometrium across eutherian mammals. Moreover, it is thought to have re-modelled the gene regulatory network of the placenta in endometrial cells [60]. Evidence that the host utilizes genes derived from TEs to develop new tissues or organs comes, for example, from the conserved *PEG10*. This is a paternally expressed imprinted gene that is highly conserved across mammals and that has a variety of functions, among which is contributing to the development of the placenta. *PEG10* is thought to be derived from a *Ty3/Gypsy* LTR retrotransposon [112]. Remarkably, *Pax6* an evolutionary conserved “master control” gene that regulates the morphogenesis of the eye, seems to have inherited its paired domain (a DNA binding region) from an ancestral transposase [113]. Gage and collaborators have shown, both in the developing brain and in adult hippocampal neurogenesis, that active LINE-1 transposons, causing somatic mosaicism, could be essential for generating the complexity of the brain [114,115,116]. Furthermore, LINE-1 retrotransposition events have been documented in cultured mouse neuronal progenitor cells, and a higher number of copies of LINE-1 were found in adult human brains than in other tissues. These results suggest that LINE-1 may play a role in neuronal plasticity [117,118]. Indeed, the LINE-1 promoter becomes activated when neural precursors differentiate into neurons and glia, whereas in stem cells, both neural transcription factors and epigenetic modifications repress the activity of TEs. This repression decreases with differentiation [117,119]. Interestingly, specific insertions in genes important for neural function have also been identified in the brain of *D. melanogaster* [120]. This finding supports the attractive hypothesis that transposition may be a conserved mechanism for neuronal plasticity, in response to environmental signals [114].

### 3.3. TEs and Chromosome Structure

TEs and derived sequences comprise 22% of the *D. melanogaster* genome [121] and roughly half of the human genome [122]. They reside primarily in heterochromatic (repressive chromatin) regions in diverse species, from flies [123] to plants [124].

There are several ways in which transposable elements may have contributed to the formation of heterochromatin. First, by gene silencing [125]. Heterochromatin proteins can recognize and silence transposon arrays when located in euchromatin (active chromatin) [126]. Therefore, the heterochromatin may have evolved through a progressive expansion of domains, rich in transposable elements.

Furthermore, it has been suggested that the first step leading to the formation and degeneration of the Y chromosome was the accumulation of transposable elements in one of two autosomes, then evolved into sex chromosomes. In flies, the neo-Y chromosome accumulated several insertion elements, especially retrotransposons, accounting for the gradual transformation of euchromatin in heterochromatin [127,128,129].

TEs actively contribute to the formation and function of centromeres and telomeres. Both structures are essential for chromosome function and genome integrity. The centromere is crucial for the segregation of chromosomes at cell division; telomeres are required for preventing chromosome shortening following replication (Figure 2e).

The telomeres of *D. melanogaster* consist of three TEs, located at the chromosome ends: *healing transposon* (*HeT-A*), *telomere associated retrotransposon* (*TART*), and *telomere associated and HeT-A related* (*TAHRE*); collectively, they are known as the “*HTT array*” [130,131,132,133,134,135,136]. These non-LTR retroelements transpose, specifically, to chromosome ends, where they are present as tandem arrays [137,138]. In flies, the stability of the telomeres is regulated by specific proteins, among which are HOAP and HIPHOP, belonging to the telomere capping complex [139,140]. Another important factor is Heterochromatin Protein 1 (HP1). HP1 binds to telomeric DNA, thus, participating in telomere capping [141]. Additionally, by interacting with methylated histone 3 at lysine 9 (H3-MeK9), it contributes to the elongation of telomeres and to the transcriptional repression of telomeric sequences [142]. In some *Drosophila* species, telomeres can carry degenerate elements unable to transpose. In such species, the stability of the telomeres is orchestrated by additional mechanisms. For instance, in *D. virilis,* the *HeT-A* transposon carries within the 3′-UTR a chimeric element, *U^vir^*, that contains a *pol* coding sequence from *Jockey,* a LINE retrotransposon [143]. *U^vir^* was the first recombinant element found in *D. virilis* telomeres and, as shown by genetic studies, its continued presence suggests recombination between telomeric arrays.

In plant genomes, TEs are not randomly distributed [144]. In rice (*Oryza sativa*), sorghum (*Sorghum bicolor*) and maize, LTRs are mainly located in heterochromatic centromeric regions, whereas DNA transposons are preferentially sited in telomeric portions [145,146]. Indeed, studies in plants suggest that recombination and rolling circle replication, mechanisms through which some DNA transposons can mobilize, may function as methods of alternative lengthening of telomere (ALT), in cases where there is loss of telomerase activity [147].

TEs also seem to be important in centromeric function. Most eukaryotes, including *Drosophila* [148], humans [149], and maize [150], have centromeres of variable size and sequence but all consisting of long tandem arrays of short repeats (satellite DNA, satDNA) and mobile elements, which probably contribute to their establishment and maintenance [129,148,151,152]. In the centromeres of *A. thaliana,* the internal satellite arrays are interspersed with retrotransposons, while the external pericentromeric region is enriched with DNA transposons [153].

Interestingly, in *Drosophila,* the telomeric transposons *Het-A* and *TART* are localized also in the centromeric heterochromatin of the Y chromosome, suggesting that centromeres may have derived from telomeric sequences [154,155]. More generally, sequence homologies between satDNA and transposons/retrotransposons have been identified in several species, which raises the possibility that satellite repeats may originate from mobile elements. For instance, in many *Drosophila* species the abundant *Minime* elements contain two internal proto-microsatellite regions, one of which can expand into long microsatellite repeats [156], such as those found in satDNA.

In addition to contributing novelty in gene function, regulatory networks, and chromosome structure, TEs have been involved in the generation of chromosome rearrangements that change the organization and the architecture of the genome [157,158].

Ectopic recombination between repeated DNA sequences has been implicated in the generation of inversions in diverse organisms, such as yeast [159], humans [160] and *Drosophila*. In *D. buzzatii,* there is a clear example of chromosome reshuffling, in the form of a wide inversion on chromosome 2, generated by recombination between opposite-oriented copies of the *Galileo* TE [158,161].

Recently, a growing number of studies have highlighted that the activity of TEs may be involved in shaping 3D chromosome structure [162]. For example, TEs of diverse families have a role in the establishment and maintenance of insulator boundaries, between so-called “topologically associated domains” (TADs) [163,164] (Figure 2f). The boundaries play a structural role in preventing the spread of heterochromatin marks to transcriptionally active regions [165,166]. Studies in mouse and human embryonic stem cells (mESC and hESC), using Hi-C analysis [167] (a technique for studying the spatial organization of chromatin), have shown that the boundary regions of TADs are enriched in SINE *Alu* retrotransposons [163]. In addition, Murine Endogenous Retroviral Elements (MERVL), which can preferentially integrate at TADs boundaries, have been shown to drive chromatin organization during zygotic genome activation in mammals [168].

Following transposition, an increase in copy number of transposable elements can result in a significant expansion in the size of the genome, over a relatively short evolutionary time. For instance, the genome of maize doubled during the last few million years [169].

In some cases, structural changes, such as inversions, resulting from the activity of TEs, can pose reproductive barriers among individuals of the same species in relatively short time and lead to speciation [151]. The P-element-mediated hybrid dysgenesis, described above, is a notable example [32,33].

## 4. Regulation of the Transposition of TEs

Summarizing, on the one hand, TEs can induce deleterious mutations, causing dysfunction, disease and even lethality in individuals. On the other hand, TEs can increase genetic variability, affording to populations a better adaptive response to environmental change. For this double effect, TEs can be considered at the same time ‘parasites’ and ‘symbionts’ of the genome, according to the modulation of their activity at the cellular level.

To counteract the deleterious effects of TEs, organisms have evolved strategies to avoid their activation and mobilization. Epigenetic mechanisms for suppressing TE mobilization are mainly based on RNA silencing and are highly conserved in eukaryotes [170]. Small non-coding RNAs (sncRNAs) degrade cytoplasmic RNA by post-transcriptional gene silencing (PTGS). Furthermore, TEs can be repressed by the formation of heterochromatin [171].

**Figure 3:**
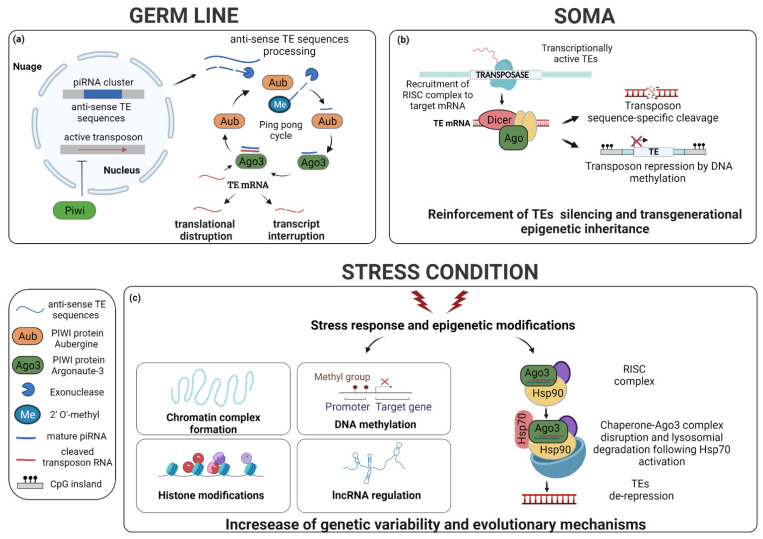
Regulation of TEs in germline and soma under standard and stress conditions. (**a**) Stepwise model of transcriptional silencing guided by Piwi-interacting RNAs (piRNAs). In the germline of *Drosophila* the silencing of TEs is guided by specific, small RNAs organized in piRNA clusters enriched in TE sequences. At the core of the pathway is the piRNA-induced silencing complex (pi-RISC) that consists of a single-stranded piRNA bound to a Piwi family protein. Piwi (light green) can guide the transcriptional silencing of TEs through direct assembly of the complex at heterochromatin target sites. *Drosophila* harbors three Piwi-like proteins: Piwi, Aubergine (Aub, orange), and Argonaute 3 (Ago3, dark green), which, guided by piRNAs, silence TEs post-transcriptionally (in addition to transcriptional silencing referred to above) through homology-dependent cleavage. Anti-sense TE sequences are exported from the nucleus and processed into smaller fragments by Aub before being loaded into Ago3. The resulting piRNA–Ago3 complexes cleave newly antisense piRNA precursors from clusters loaded into Aub to produce anti-sense piRNAs, resulting in a “ping-pong” amplification cycle. Additionally, Aub–piRNA complexes can bind TE transcripts and repress their translation directly. (**b**) In the soma the repression of TEs is mediated predominantly by the small interfering RNA (siRNA) pathway. siRNA precursors form short hairpin structures before being processed and incorporated into the RNA-induced silencing complex (RISC). Once a siRNA binds to its target TE mRNA, it induces the cleavage of such an mRNA by RISC. Other silencing mechanisms involve chromatin remodeling. Several inhibitory marks, such as DNA methylation and histone methylation and deacetylation play an important role in repressing the mobilization of TEs. These epigenetic modifications may be passed on by dividing cells from one generation to the next. (**c**) When individuals are exposed to drastic environmental changes, they may experience stress-induced (re)activation of TEs. Epigenetic mechanisms like DNA methylation, histone modifications, expression of non-coding RNAs (ncRNAs) seem particularly relevant for this phenomenon. Additionally, environmental stress, such as heat shock, can induce de-repression of TEs by inducing the disruption of RISC through the action of the inducible Hsp70 chaperone, which targets the complex to the lysosome. The generalized reactivation of TEs can generate genome instability leading to higher risk of disease when occurring in somatic cells and to infertility when arising in germ cells. However, in the latter it brings about increased genetic variability also, which is key for an adaptive response to extreme environmental change.

In the *Drosophila* germline, transposable elements and other repetitive sequences are repressed by Piwi-interacting RNAs (piRNAs) [172,173] (Figure 3a), whose expression is mediated by the heat shock 90 (HSP90) protein [174]. Importantly, in *Drosophila* mutants defective for piRNA biogenesis, p53 is constitutively active. Further confirmation comes from the findings that *TAHRE* elements, known targets of piRNAs, are strongly upregulated in the ovaries of p53-null flies but not in transgenic flies, whose p53 activity is rescued [175]. Additionally, the ovaries of *Drosophila* and *zebrafish* p53 mutants show up-regulation of *Idefix*, *Burdock*, and *Gypsy* retroelements. These data suggest that the repression of retrotransposition requires the interaction between p53 and piRNA pathways [176].

In the somatic cells of flies, small interfering RNAs (siRNA) are main players in the repression of transposons via the direct RNA interference (RNAi) mechanism (Figure 3b). As demonstrated by Ghildiyal et al., some endo-siRNAs, most probably derived from exogenous double-stranded RNA (dsRNA), can silence TEs through the synergistic action of the ribonucleases Dicer-2 and Argonaute 2 (Ago2) [177].

In *A. thaliana*, retrotransposons are inactivated by DNA methylation, following an increase in their copy number. In fact, in *ddm* DNA methylation mutants TEs are reactivated [178,179]. Such an outcome (reactivation of retroelements due to the lack of DNA methylation) occurs also in mice, suggesting that this mechanism may have evolved as a basic defense to prevent the harmful activity of mobile elements [90,180,181,182].

In the human genome, LINE-1 elements are the most abundant family of transposons. As for other elements and other organisms, *LINE-1* are silenced through the concerted action of several mechanisms, amongst which are the piRNA pathway, DNA methylation and histone modifications [183]. For instance, MORC1, encoded by the founder member of the *Morc* gene family *Morc1* [184], represses *LINE1* (and *IAP* retrotransposons) via modulation of DNA methylation [181,185].

APOBEC (Apolipoprotein B mRNA Editing Catalytic Polypeptide-like) proteins control *LINE-1* integration [186,187], although the mechanism is not fully understood [188]. The Microprocessor complex (Drosha/DGCR8), a nuclear complex implicated in microRNA (miRNA) biosynthesis, restraints *LINE-1* abundance through cleavage-dependent degradation [189,190]. The longevity-regulating protein SIRT6 intervenes also by binding to *LINE-1* promoters and mono-ADP ribosylating the nuclear corepressor protein KRAB-associated protein 1 (KAP1), causing chromatin compaction. Under conditions of stress, SIRT6 relocates to DNA damage sites, removing an important obstacle to *LINE-1* mobilization [191]. Additionally, some of these pathways mediate the deposition of chromatin modification marks, mostly histone tail modifications, such as H3K9- and H4K20-trimethylation [90,192], causing further silencing.

Although TEs are maintained silent through these mechanisms, they can reactivate under some conditions. For example, studies have shown that some TEs become highly expressed during short temporal windows of germline development. Perhaps, the cycles of replication occurring in these cells could lead to TE mobilization. This also raises the possibility that TE transcripts, produced at these specific stages, may play a cellular role during early development [193].

In the *Drosophila* germline, TEs are transcribed, but the piRNA pathway blocks their activity post-transcriptionally. Conversely, we know little about relaxation of silencing that may occur during embryonic, larval, and pupal development. Marie et al. reported that P-element repression might be occasionally relaxed due to incomplete silencing, established in embryonic germ cells and stably maintained throughout development [194]. Beside such early de-repression, a spatiotemporal window, named the “PiwiLess Pocket” (Pilp), exists in the dividing cysts of adult ovaries, during which TEs can escape silencing from the host [193].

De-repression of TEs can occur because of external changes (Figure 3c). McClintock discussed the phenomenon of TE activation and transposition during stress, suggesting that, as a result, rearranged genomes may induce the formation of new species [14]. Evidence shows that some TEs are expressed and/or mobilized under stress [191,195,196,197,198,199,200,201]. TEs are known to be expressed in response to biotic factors, such as competition, predation and parasitism [202,203,204], and abiotic factors, such as heat shock, DNA damage, UV radiation, climate, and chemical compounds [205,206,207,208,209,210,211,212,213,214].

Several studies have identified molecular mechanisms that cause the activation of TEs under stress. As mentioned above, while *LINE1s* are silenced by SIRT6 under normal conditions, under stress, this protein relocates to DNA damage sites, providing an opportunity for activation [191].

Under conditions of stress, the insertion of TEs is often associated with upregulation of nearby genes [215]. In the mouse genome, the chaperone heat-shock protein HSP90 forms a complex with KAP1 that binds to *ERVs*. Following stress, the function of the HSP90–KAP1 complex is compromised and, as a result, *ERVs* located in gene regulatory regions drive the expression of nearby genes [199]. In *Drosophila* germ cells, heat shock increases the expression of TEs, mainly at the post-transcriptional level, by affecting piRNA biogenesis through the action of the HSP70 chaperone. The interaction of HSP70 with the HSC70-HSP90 complex and other factors induces their displacement to the lysosome and their degradation, resulting in the decrease in piRNA biogenesis [216].

## 5. Environmental Stress and Evolution

As predicted by Barbara McClintock, TEs are not only able to clarify some aspects of the temporal dynamics of evolution. Additionally, they can also explain how genetic variability, which is necessary for the adaptation of living organisms to a changing environment, is produced. In a changing environment, organisms are faced with three possibilities: to move to a different geographic area, to adapt to the different environmental conditions [217] or to become extinct. Adaptation to new environments can occur either through phenotypic plasticity or through induction of genetic variability. In both cases, the resulting phenotypes must pass the sieve of natural selection. Phenotypic plasticity is defined as the ability for a genotype to express several phenotypes, according to different environmental cues [218,219]. Plastic responses represent the initial morphological, physiological or behavioral answer to environmental change [220], but to be adaptive, the ability to give plastic responses has to be transmitted. Evolutionary responses occur across generations, and the rate at which populations can evolve depends on the strength of selection and on the amount of genetic variation [221].

Several reports highlight the role of TEs in inducing genetic variability and adaptation [222,223,224,225]. Recently, a new evolutionary mechanism has been proposed within a Darwinian framework. It is based on the ability of the environment to induce genetic variability through the expression and mobilization of TEs, following stress [198,226]. This model results from experiments aimed at verifying the validity of Conrad Waddington’s theory of “canalization and assimilation” [227,228,229], reviewed and revisited in [230,231,232,233]. Waddington tried to explain how a population could inherit a trait acquired in response to an environmental stimulus, without falling into a Lamarckian scenario. He introduced the concept of “epigenetic landscape” where organisms (exemplified as spheres/cells), glide through valleys and ridges, following a random trajectory that he defined a “creode”, a “necessary path”. Trajectories indicate the set of phenotypes that a given genotype can produce when exposed to different environments during development. Such a theory is based on the idea of pre-existing cryptic genetic variability within populations, kept hidden by the robustness of the developmental processes (canalization), through a buffer system, later identified as the HSP90 chaperone [234]. However, such a cryptic variability would manifest itself following specific environmental conditions. Waddington exposed *Drosophila* flies at the pupal stage to heat shock. He observed that some individuals, once adults, showed morphological anomalies of the posterior veins of the wing, thus, simulating the “crossveinless” mutation. The explanation, under a phylogenetic and ontogenetic scenario of the epigenetic landscape theory, is that the presence of a selective pressure would favor one trajectory over the others, allowing the expression of a new phenotypes. Subsequently, Waddington heat shocked pupae at every generation and selected individuals with the same morphological anomalies. Eventually, he obtained individuals with the wing anomaly also in the absence of heat shock. His interpretation was that the expression of this character, and not the character itself (because it was already present), had been fixed in the genotype, through a mechanism that he named “genetic assimilation” [228,229].

Recently, a different explanation of the results by Waddington has been proposed. Individuals from natural populations of *Drosophila* that were subject to heat stress as pupae, as done by Waddington, often presented morphological anomalies when adults. In principle, these variants could represent epigenetic modifications or genetic mutations. Epigenetic modifications follow heat shock and change the activation status of genes, inducing phenocopies. However, among the variants discovered, some of them continued to manifest and to be transmitted across generations, even in the absence of stress, showing to be true mutations. When characterized molecularly, they appeared to be caused by the insertion of transposons into coding genes. The concomitant appearance of phenocopies and true mutations with the same phenotype is interpreted as a process of co-selection or pseudo-assimilation of genetic variants [198]. There is a correlation between the insurgence of epigenetic modifications and transposition [235]. Thus, the phenomenon of co-selection between phenocopies and true mutations with the same phenotype is probably more frequent than one may imagine at first. Indeed, not only TEs can modify the epigenetic landscape at the insertion site with repercussions on gene expression and the production of a phenocopy. Additionally, changes in chromatin can create preferential capture site for TEs, inducing new mutations.

Importantly, such mechanisms allow correlating environmental stresses with HSP-mediated activation of TEs, resulting in the generation of genetic variability that underlies evolution [198]. To explain: in *Drosophila*, HSP70 is stress-inducible and plays a key role in protecting individuals and populations. It does so by increasing cell survival throughout its chaperone function [236], but also by increasing the frequency of mutations in the germline [216,226]. The induction of HSP70, following stress, is precisely regulated. The transcription of the *Hsp70* gene is activated by heat-shock factors (HSFs) that recognize unfolded proteins in the cells [237,238,239,240]. However, transcription becomes rapidly attenuated when stress conditions decline, to mitigate the influence that HSP70 has on cell growth and division [241,242]. Therefore, it is conceivable that the transcription of *Hsp70* recapitulates the environmental stress that organisms experience [243,244]. Once produced, HSP70 interacts with HSP90 “distracting” it from its piwi-RNAs regulatory function and allowing TE expression [216]. It follows that the severity of the stress, which is reflected by the level of expression of HSP70, modulates the consequent activation of TEs.

This is a well-documented example of how environmental change can drive the insurgence of genetic variability, specifically, by hitchhiking the complex regulation of HSPs and inducing a relaxation in the repression of TEs. Despite such a mechanism being demonstrated in *Drosophila,* limited to laboratory conditions, currently, many laboratories, including ours, are testing this phenomenon in natural populations of *Drosophila* and in additional species. These investigations are important and timely and will help us understand the effects that environmental change may have on natural populations.

## 6. Conclusions

Transposons modify the architecture of genomes and influence evolutionary processes. The fine balance in their repression and activation affects both the temporal dynamics of evolution and the production of genetic variability. At the level of populations, it appears that TEs may become more active when it is advantageous to increase genetic variability; that is, when the environmental changes are such that the previous adaptations are no longer effective [222]. In principle, the TE-mediated stress response is not only adaptive, but also evolvable [245]. In fact, only populations capable of modulating the intensity of the response to environmental conditions would be positively selected and will be resistant to extinction. Populations that give too weak or too high a response would succumb, because they would not be able to induce sufficient genetic variability (the former) or because they will be overwhelmed by excessive mutagenesis (the latter) [226]. We speculate that the ability of TEs to increase genetic variability, by causing mutation, following harsh environmental conditions, may be a common phenomenon across species. If proven true, one interesting evolutionary consequence would be that several genetic diseases found in human populations may be considered collateral damage [246] that falls on individuals, because of a mechanism that has evolved to protect species from extinction.
